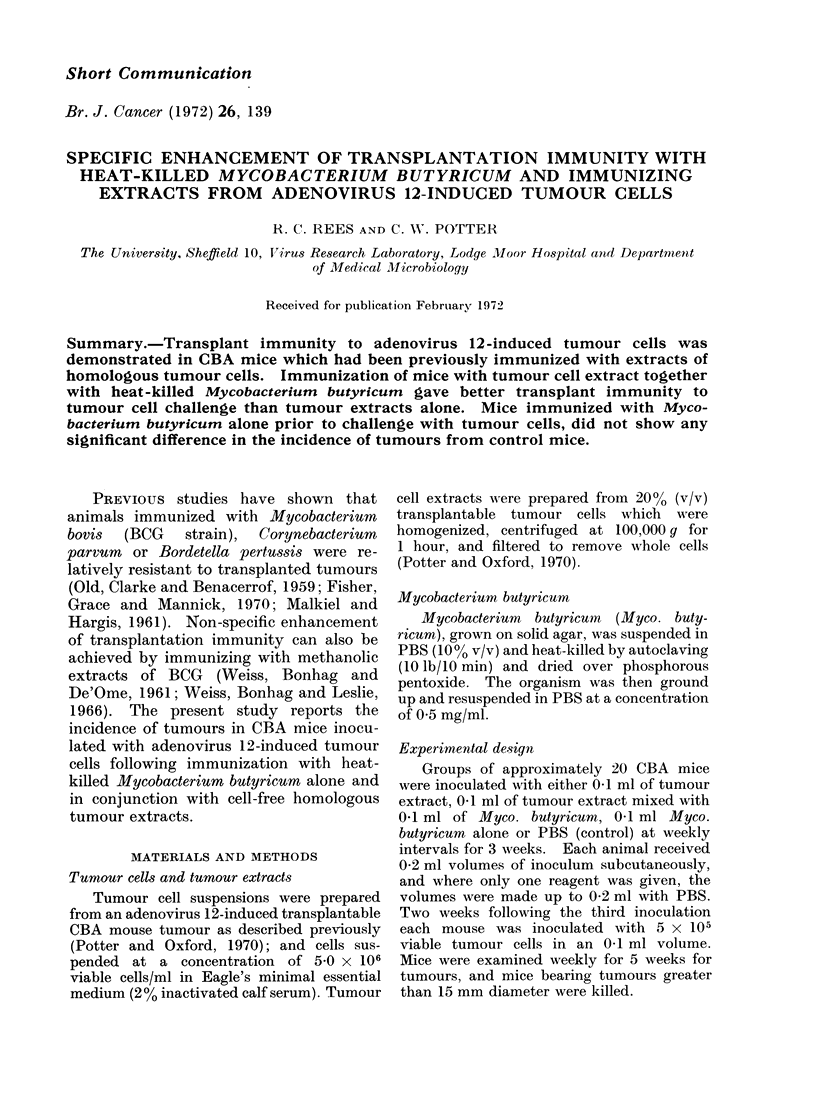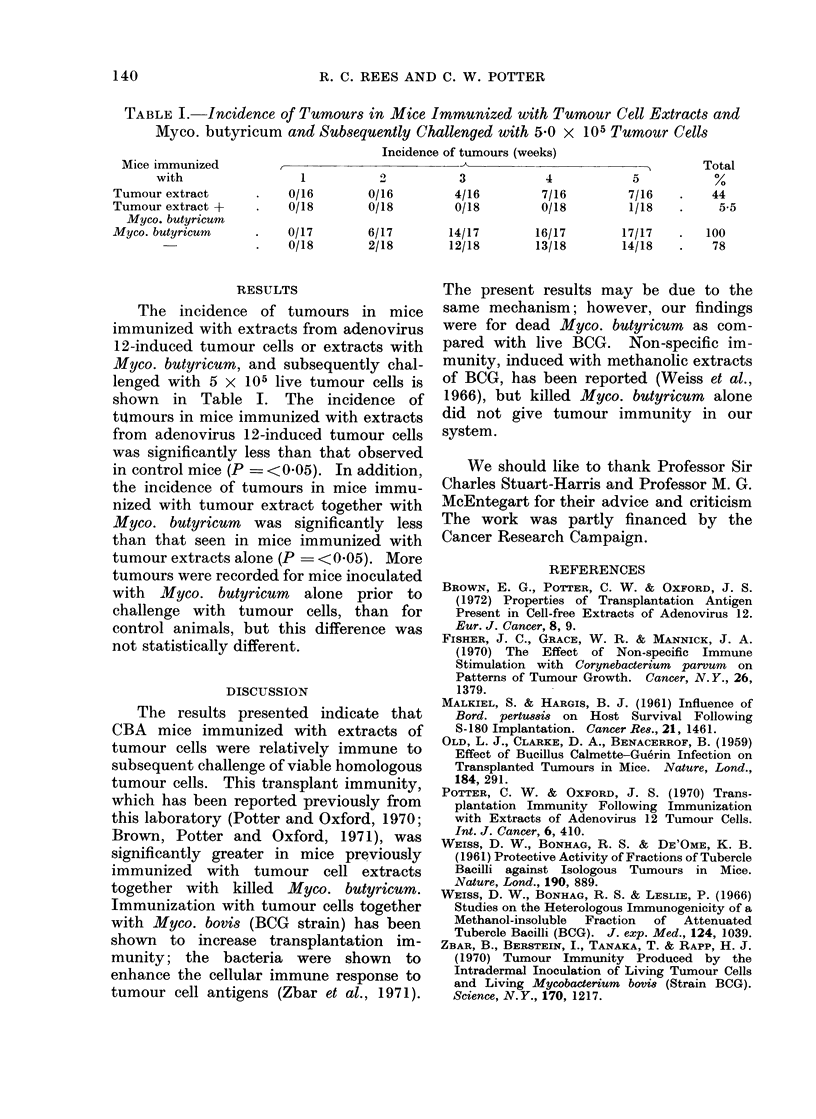# Specific Enhancement of Transplantation Immunity with Heat-killed Mycobacterium Butyricum and Immunizing Extracts from Adenovirus 12-Induced Tumour Cells

**DOI:** 10.1038/bjc.1972.20

**Published:** 1972-04

**Authors:** R. C. Rees, C. W. Potter

## Abstract

Transplant immunity to adenovirus 12-induced tumour cells was demonstrated in CBA mice which had been previously immunized with extracts of homologous tumour cells. Immunization of mice with tumour cell extract together with heat-killed *Mycobacterium butyricum* gave better transplant immunity to tumour cell challenge than tumour extracts alone. Mice immunized with *Mycobacterium butyricum* alone prior to challenge with tumour cells, did not show any significant difference in the incidence of tumours from control mice.


*Short Communication*

*Br. J. Cancer* (1972) **26**, 139

### SPECIFIC ENHANCEMENT OF TRANSPLANTATION IMMUNITY WITH HEAT-KILLED *MYCOBACTERIUM BUTYRICUM* AND IMMUNIZING EXTRACTS FROM ADENOVIRUS 12-INDUCED TUMOUR CELLS

R. C. REES AND C. W. POTTER

*The University, Sheffield* 10, *Virus Research Laboratory, Lodge Moor Hospital and Department of Medical Microbiology*

Received for publication February 1972

**Summary.—Transplant immunity to adenovirus 12-induced tumour cells was demonstrated in CBA mice which had been previously immunized with extracts of homologous tumour cells. Immunization of mice with tumour cell extract together with heat-killed *Mycobacterium butyricum* gave better transplant immunity to tumour cell challenge than tumour extracts alone. Mice immunized with *Mycobacterium butyricum* alone prior to challenge with tumour cells, did not show any significant difference in the incidence of tumours from control mice.**

PREVIOUS studies have shown that animals immunized with *Mycobacterium bovis* (BCG strain), *Corynebacterium parvum* or *Bordetella pertussis* were relatively resistant to transplanted tumours (Old, Clarke and Benacerrof, 1959; Fisher, Grace and Mannick, 1970; Malkiel and Hargis, 1961). Non-specific enhancement of transplantation immunity can also be achieved by immunizing with methanolic extracts of BCG (Weiss, Bonhag and De'Ome, 1961; Weiss, Bonhag and Leslie, 1966). The present study reports the incidence of tumours in CBA mice inoculated with adenovirus 12-induced tumour cells following immunization with heat-killed *Mycobacterium butyricum* alone and in conjunction with cell-free homologous tumour extracts.

### MATERIALS AND METHODS

### *Tumour cells and tumour extracts*

Tumour cell suspensions were prepared from an adenovirus 12-induced transplantable CBA mouse tumour as described previously (Potter and Oxford, 1970); and cells suspended at a concentration of $5{\cdot}0 \times 10^6$ viable cells/ml in Eagle's minimal essential medium (2% inactivated calf serum). Tumour cell extracts were prepared from 20% (v/v) transplantable tumour cells which were homogenized, centrifuged at 100,000 *g* for 1 hour, and filtered to remove whole cells (Potter and Oxford, 1970).

### *Mycobacterium butyricum*

*Mycobacterium butyricum* (*Myco. butyricum*), grown on solid agar, was suspended in PBS (10% v/v) and heat-killed by autoclaving (10 lb/10 min) and dried over phosphorous pentoxide. The organism was then ground up and resuspended in PBS at a concentration of 0·5 mg/ml.

### *Experimental design*

Groups of approximately 20 CBA mice were inoculated with either 0·1 ml of tumour extract, 0·1 ml of tumour extract mixed with 0·1 ml of *Myco. butyricum*, 0·1 ml *Myco. butyricum* alone or PBS (control) at weekly intervals for 3 weeks. Each animal received 0·2 ml volumes of inoculum subcutaneously, and where only one reagent was given, the volumes were made up to 0·2 ml with PBS. Two weeks following the third inoculation each mouse was inoculated with $5 \times 10^5$ viable tumour cells in an 0·1 ml volume. Mice were examined weekly for 5 weeks for tumours, and mice bearing tumours greater than 15 mm diameter were killed.

TABLE I.—*Incidence of Tumours in Mice Immunized with Tumour Cell Extracts and* Myco. butyricum *and Subsequently Challenged with* $5 \cdot 0 \times 10^5$ *Tumour Cells*

| Mice immunized with | Incidence of tumours (weeks) 1 | 2 | 3 | 4 | 5 | Total % |
|---|---|---|---|---|---|---|
| Tumour extract | 0/16 | 0/16 | 4/16 | 7/16 | 7/16 | 44 |
| Tumour extract + *Myco. butyricum* | 0/18 | 0/18 | 0/18 | 0/18 | 1/18 | 5·5 |
| *Myco. butyricum* | 0/17 | 6/17 | 14/17 | 16/17 | 17/17 | 100 |
| — | 0/18 | 2/18 | 12/18 | 13/18 | 14/18 | 78 |

### RESULTS

The incidence of tumours in mice immunized with extracts from adenovirus 12-induced tumour cells or extracts with *Myco. butyricum*, and subsequently challenged with $5 \times 10^5$ live tumour cells is shown in Table I. The incidence of tumours in mice immunized with extracts from adenovirus 12-induced tumour cells was significantly less than that observed in control mice ($P = < 0 \cdot 05$). In addition, the incidence of tumours in mice immunized with tumour extract together with *Myco. butyricum* was significantly less than that seen in mice immunized with tumour extracts alone ($P = < 0 \cdot 05$). More tumours were recorded for mice inoculated with *Myco. butyricum* alone prior to challenge with tumour cells, than for control animals, but this difference was not statistically different.

### DISCUSSION

The results presented indicate that CBA mice immunized with extracts of tumour cells were relatively immune to subsequent challenge of viable homologous tumour cells. This transplant immunity, which has been reported previously from this laboratory (Potter and Oxford, 1970; Brown, Potter and Oxford, 1971), was significantly greater in mice previously immunized with tumour cell extracts together with killed *Myco. butyricum*. Immunization with tumour cells together with *Myco. bovis* (BCG strain) has been shown to increase transplantation immunity; the bacteria were shown to enhance the cellular immune response to tumour cell antigens (Zbar *et al.*, 1971). The present results may be due to the same mechanism; however, our findings were for dead *Myco. butyricum* as compared with live BCG. Non-specific immunity, induced with methanolic extracts of BCG, has been reported (Weiss *et al.*, 1966), but killed *Myco. butyricum* alone did not give tumour immunity in our system.

We should like to thank Professor Sir Charles Stuart-Harris and Professor M. G. McEntegart for their advice and criticism The work was partly financed by the Cancer Research Campaign.

### REFERENCES

BROWN, E. G., POTTER, C. W. & OXFORD, J. S. (1972) Properties of Transplantation Antigen Present in Cell-free Extracts of Adenovirus 12. *Eur. J. Cancer*, **8**, 9.

FISHER, J. C., GRACE, W. R. & MANNICK, J. A. (1970) The Effect of Non-specific Immune Stimulation with *Corynebacterium parvum* on Patterns of Tumour Growth. *Cancer, N.Y.*, **26**, 1379.

MALKIEL, S. & HARGIS, B. J. (1961) Influence of *Bord. pertussis* on Host Survival Following S-180 Implantation. *Cancer Res.*, **21**, 1461.

OLD, L. J., CLARKE, D. A., BENACERROF, B. (1959) Effect of Bucillus Calmette–Guèrin Infection on Transplanted Tumours in Mice. *Nature, Lond.*, **184**, 291.

POTTER, C. W. & OXFORD, J. S. (1970) Transplantation Immunity Following Immunization with Extracts of Adenovirus 12 Tumour Cells. *Int. J. Cancer*, **6**, 410.

WEISS, D. W., BONHAG, R. S. & DE'OME, K. B. (1961) Protective Activity of Fractions of Tubercle Bacilli against Isologous Tumours in Mice. *Nature, Lond.*, **190**, 889.

WEISS, D. W., BONHAG, R. S. & LESLIE, P. (1966) Studies on the Heterologous Immunogenicity of a Methanol-insoluble Fraction of Attenuated Tubercle Bacilli (BCG). *J. exp. Med.*, **124**, 1039.

ZBAR, B., BERSTEIN, I., TANAKA, T. & RAPP, H. J. (1970) Tumour Immunity Produced by the Intradermal Inoculation of Living Tumour Cells and Living *Mycobacterium bovis* (Strain BCG). *Science, N.Y.*, **170**, 1217.